# Gold nanoparticle-assisted all optical localized stimulation and monitoring of Ca^2+^ signaling in neurons

**DOI:** 10.1038/srep20619

**Published:** 2016-02-09

**Authors:** Flavie Lavoie-Cardinal, Charleen Salesse, Éric Bergeron, Michel Meunier, Paul De Koninck

**Affiliations:** 1 Institut Universitaire en Santé Mentale de Québec, 2601 de la Canardière, Québec, QC, G1J 2G3, Canada; 2 Département de Biochimie, Microbiologie et Bio-informatique, Université Laval, Québec, QC, G1V 0A6, Canada; 3 Laser Processing and Plasmonics Laboratory, Engineering Physics Department, École Polytechnique de Montréal, Montréal, QC, H3C 3A7, Canada

## Abstract

Light-assisted manipulation of cells to control membrane activity or intracellular signaling has become a major avenue in life sciences. However, the ability to perform subcellular light stimulation to investigate localized signaling has been limited. Here, we introduce an all optical method for the stimulation and the monitoring of localized Ca^2+^ signaling in neurons that takes advantage of plasmonic excitation of gold nanoparticles (AuNPs). We show with confocal microscopy that 800 nm laser pulse application onto a neuron decorated with a few AuNPs triggers a transient increase in free Ca^2+^, measured optically with GCaMP6s. We show that action potentials, measured electrophysiologically, can be induced with this approach. We demonstrate activation of local Ca^2+^ transients and Ca^2+^ signaling via CaMKII in dendritic domains, by illuminating a single or few functionalized AuNPs specifically targeting genetically-modified neurons. This NP-Assisted Localized Optical Stimulation (NALOS) provides a new complement to light-dependent methods for controlling neuronal activity and cell signaling.

Light-based stimulation of neurons or neuronal networks with optogenetic tools has become a well-established and powerful approach to study neural circuit function^1^. It provides several advantages over electrical stimulation, such as the capacity to genetically control the subtype of neurons to be stimulated, the capacity to excite or inhibit neuronal activity and in some cases, a reduced level of invasiveness^2^. However, to investigate local signaling processes, triggered by local membrane activity, optogenetics has not been successful, primarily because light-gated channels have a very small conductance. Neurotransmitter uncaging, with ultraviolet (UV) or two-photon excitation, has been elegantly used to drive single synapse activation^3^. The precision of uncaging is very high, such that the laser beam must be within 1 μm of an identified synapse, usually a dendritic spine (recognizable by its shape) to evoke a synaptic response. This approach, however, cannot be used to activate a dendritic response at a non-synaptic site. On the other hand, infrared (IR) light-induced neural stimulation with short laser pulses has been shown to excite neurons by affecting membrane capacitance, likely via a local heating process^4^,^5^. Moreover, femtosecond (fs) laser pulses could be used successfully for targeted gene transfection and Ca^2+^ wave induction^6^,^7^,^8^,^9^,^10^. Interestingly, gold nanoparticles (AuNP)-assisted IR neural stimulation using gold nanorods has been demonstrated to increase neurite growth and whole cell electrical response^11^,^12^,^13^. AuNPs are known to convert light into heat after excitation with a visible continuous wave (cw) laser^14^,^15^. Upon excitation of AuNPs with fs laser pulses, near-field enhancement and plasma generation was previously related to nanocavitation formation and could be used successfully for AuNP-assisted cell perforation and transfection^16^,^17^,^18^,^19^. Characterization of the phenomenon with simulation and experimental data was performed for a fs laser operating at 1 kHz, but due to pileup effects, the exact mechanism taking place for higher repetition rates remains unclear^19^. Recently, it was shown that irradiating functionalized 20 nm AuNPs by a 1 ms 532 nm light pulse at a repetition rate of 40 Hz could be used to trigger action potentials (APs)^14^. However, up to now, targeted localized stimulation of single AuNPs and the corresponding subcellular response have not been demonstrated.

Here, we present a technique called Nanoparticle-Assisted Localized Optical Stimulation (NALOS) for localized, diffraction-unlimited stimulation of subcellular regions on cultured hippocampal neurons by the mean of plasmonic excitation of single AuNPs. To combine optical stimulation and optical read-out of neuronal activity, we used the off-resonance plasmonic excitation of AuNPs with near-IR light (NIR) at 800 nm as a source for localized neural stimulation, and optical imaging of Ca^2+^ oscillations, using a standard commercial confocal microscope. While non-specific binding of bare AuNPs onto the neurons was possible through passive sedimentation, we show that AuNP functionalization with monoclonal antibodies can be used to specifically target their localization on neuronal membranes expressing the targeted antigen. We demonstrate with combined Ca^2+^ imaging and whole-cell patch-clamping that photo-stimulation of neurons decorated with bare or functionalized AuNPs can drive local or widespread Ca^2+^ responses and APs, depending on the intensity and localization of the illumination. Finally, we demonstrate that NALOS can be used to investigate local Ca^2+^ signaling in dendritic compartments, by monitoring the spatial dynamics of Ca^2+^/calmodulin-dependent protein kinase II (CaMKII).

## Results

### Nanoparticle-assisted localized optical stimulation with bare gold nanoparticles

**Figure 1:**
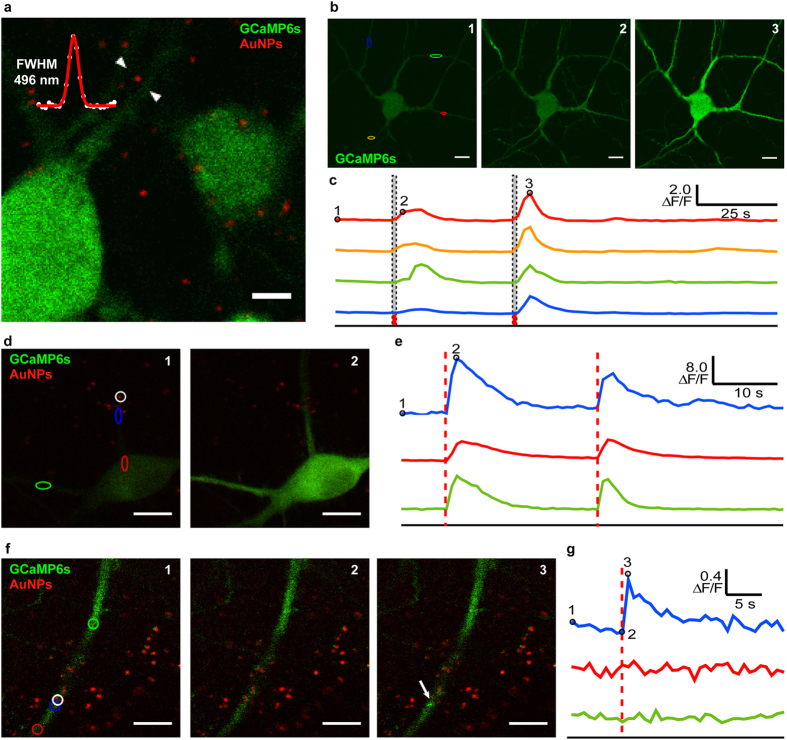
Neuronal stimulation with illumination of bare AuNPs. (**a**) Fluorescence of GCaMP6s (green) and reflectance at 800 nm of the AuNPs (red). The measured full width at half maximum (FWHM) of the reflection PSF of a single AuNP is shown in the upper left corner. (**b**) Representative example from 21 neurons of the change in GCaMP6s fluorescence following a single scan of the whole imaging field with the NIR fs laser, at the time points indicated by the dotted lines in (**c**). The labels “1, 2, 3” indicate the timing of the images corresponding to those in (**c**). (**c**) Ca^2+^ signal (ΔF/F) over time in the respective colored regions marked in (**b**); red dotted lines indicate time of laser stimulation. (**d**,**e**) NALOS in a dendrite can trigger a cell-wide Ca^2+^ transient. (**d**) Fluorescence of GCaMP6s and reflectance of the AuNPs (red) prior to (1) and after (2) NALOS in the white circle. (**e**) Ca^2+^ signal (ΔF/F) over time in the respective colored regions marked in (**d**); red dotted lines indicate timing of laser stimulation. (**f**,**g**) NALOS with lower intensity in a dendrite can trigger highly localized Ca^2+^ transient. Same approach as in (**d**,**e**). Scale bars 10 μm. More detailed quantification of the observed Ca^2+^ after NALOS is shown in Suppl. Fig. 1.

We first tested whether neurons decorated with 100 nm bare AuNPs could be stimulated by applying brief pulses of laser light. We incubated AuNPs directly onto cultured hippocampal neurons for 2h, expecting that some AuNPs would settle on their membrane via sedimentation. We reasoned that light stimulation at 800 nm should induce an off-resonance plasmonic excitation of the AuNPs, which might then serve to stimulate the neurons^18^,^19^,^20^. To monitor the neuronal response, we performed Ca^2+^ imaging with laser scanning confocal microscopy (488 nm) on neurons transfected with the Ca^2+^ sensitive protein GCaMP6s^21^. Both laser beams were focused through the same objective of a LSM-510 Meta (Zeiss) microscope. We first determined the extent of binding and the positions of the AuNPs by detecting their reflection following low intensity illumination (50 W/cm^2^ at the focus of the objective) with fs pulsed 90 MHz laser at 800 nm (Fig. 1a). Several AuNPs appeared to adhere to the neuronal membrane, remaining in place after 10 min of HEPES-based aCSF perfusion. We measured the reflection point spread function (PSF) of the AuNPs, in order to stimulate only those PSFs under 510 nm (0.8 numerical aperture (NA)) or 410 nm (1.0 NA), thus avoiding the stimulation of large AuNP aggregates.

To produce off-resonance plasmonic excitation of the AuNPs on a neuron with the 800 nm fs pulses, we first scanned the entire field of view with a laser intensity of 0.27 MW/cm^2^, while simultaneously monitoring the GCaMP6s fluorescence. A single scan of the whole field of view with the fs laser induced widespread Ca^2+^ waves in the neurons (21 of 23 neurons tested) (Fig. 1b,c). At this laser intensity, repeated stimulation could be evoked. We next applied fs laser illumination at slightly higher intensity (0.53 MW/cm^2^) to sub-regions on the soma or proximal dendrites of the neurons where a diffraction-limited single AuNP reflection spot was localized. We also observed transient widespread Ca^2+^ elevation, although the delay and amplitude varied with the location of the light stimulus (Fig. 1d,e). Altogether, we observed a reversible Ca^2+^ response following NALOS in 80% of the tested neurons, which was localized to a subdendritic region for 46% of the tested neurons (35 neurons, 3 independent cultures, Suppl. Fig. 1). Although large AuNP aggregates were excluded, single AuNPs or small AuNP clusters, non-distinguishable due to diffraction-limited PSF, may induce variable near-field enhancement and thus variability of the Ca^2+^ response for a given laser intensity. Indeed, 9% of the tested neurons showed a persistent Ca^2+^ increase after NALOS, which was most likely caused by the stimulation of a AuNP aggregate (Suppl. Fig. 1). We repeated the fs scanning over the entire field or in subregions of neurons that were not pre-incubated with AuNPs and observed no change in Ca^2+^ activity (24 cells, 5 independent cultures, Suppl. Fig. 1). These results suggest that the presence of AuNPs on the surface of the neuron was sufficient to induce Ca^2+^ elevation in neurons exposed to fs pulsed laser light at 800 nm. Since the intensity range employed for NALOS is about one order of magnitude lower than the one used for two-photon microscopy, no cross-talk was observed between stimulation and fluorescence excitation.

We next tested whether it was possible to induce a highly localized Ca^2+^ elevation by applying irradiation over a very small region of interest (ROI) around one or few AuNPs. We observed an increase in GCaMP6s fluorescence directly after scanning over an ROI (5 μm^2^) with the 800 nm fs laser at an intensity of 0.3 MW/cm^2^ (Fig. 1f). The Ca^2+^ elevation was transient and highly localized (Fig. 1g) around the ROI. These results indicate that fs illumination in NIR range of a single AuNP or a small AuNP cluster containing few AuNPs (with a diffraction-limited PSF) can induce localized Ca^2+^ transient in neurons.

### NALOS with functionalized gold nanoparticles

To enable the binding of AuNPs to specific neurons or membrane targets, we functionalized the AuNPs with monoclonal antibodies against a hemagglutinin (HA) tag, using our recently optimized protocol^22^. Indeed, our recent study showed that AuNPs functionalized with mixed layers of poly(ethylene glycol) (PEG) and receptor antibodies attached, mostly as single particles, 115 times more to receptor antigen-expressing cells compared to control cells not expressing the antibody target. Furthermore, scanning electron microscopy observations indicated that functionalized AuNPs (fAuNPs) do not form large aggregates in the cell culture medium containing serum proteins^22^. Here we compared the absorbance of AuNP samples in a spectrophotometer following or not functionalization with PEG and anti-HA antibodies, purification, and their resuspension in water or saline. The maximum of the plasmon band for bare AuNPs in water was 565 nm; according to the Mie Theory^23^, this value indicates AuNP sizes centered around 96 nm (Suppl. Fig. 2 and Supplementary Table 1)^23^. The fAuNPs exhibited a ~3 nm red-shift of the plasmon band in water or saline, consistent with their expected slightly larger size. Supplementary Fig. 2 also shows that functionalized and purified AuNPs remained mostly single and stable in physiological medium, more so than bare ones.

We chose to use an antibody against a tag (HA), rather than an endogenous receptor, to ensure specificity in the binding, which can be tested by comparing binding on neurons transfected or not with an HA-tagged receptor gene. Neurons were transfected with an HA-GluA1 subunit of the AMPA receptor, which we have used previously with HA-tagged quantum dots to track receptor mobility^24^. The neurons were also co-transfected with GCaMP6s, allowing to examine not only the Ca^2+^ response, but also to determine visually the specificity of the binding of fAuNPs and targeted cells.

**Figure 2:**
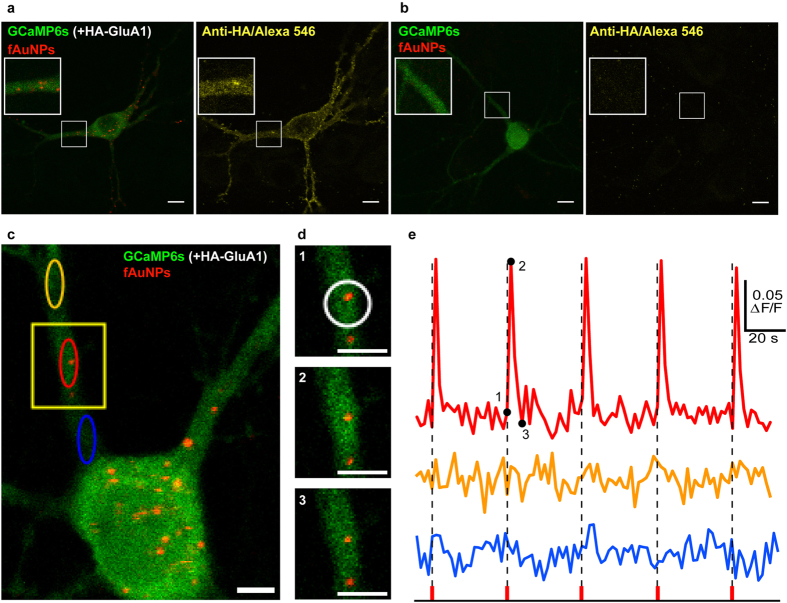
NALOS with functionalized AuNPs. (**a**,**b**) Representative neurons expressing (**a**) HA-GluA1 and GCaMP6s (n = 5) or (**b**) only GCaMP6s (n = 5) and incubated 90 min with fAuNPs, imaged as in Fig. 1. The right panels in (**a**) and (**b**) show the same neuron as on their left, following fixation and immunostaining for the Ha-tag (revealed with Alexa 546). Without the presence of the HA-GluA1, the fAuNPs (functionalized with monoclonal anti-HA antibodies) did not bind on the neurons. (**c-e**) NALOS with fAuNPs triggered localized and repeatable Ca^2+^ transients (n = 10). (**c**) Other representative neuron transfected and imaged as in (**a**). (**d**) Magnification of the region marked in (**c**) for three consecutive time points (marked with 1, 2 and 3 on the graph in (**e**)); NALOS was applied on the region marked with a white circle. (**e**) Ca^2+^ signal (ΔF/F) of the corresponding colored oval regions marked in (**c**), before and after NALOS at the time points marked by dotted lines. Scale bars (**a**,**b**) 10 μm and (**c**,**d**) 5 μm.

Following incubation and wash of the fAuNPs, we observed that their reflectance at 800 nm co-localized very specifically with the transfected fluorescent neurons, further confirmed by immunostaining the neurons with anti-HA antibodies (Fig. 2a,b and Suppl. Fig. 3). Furthermore, the aggregation phenomenon observed with bare AuNPs was clearly reduced, and the fAuNPs bound to the surface of the neurons were mobile, as observed before for GluA1 diffusing in the plasma membrane^24^. Thus, we continually monitored the position of the fAuNPs during the imaging to ensure that the localized stimulation remained on target on the transfected cells. The combination of fAuNPs with NALOS enabled the induction of repeated Ca^2+^ responses, confined around a single fAuNP, since no Ca^2+^ response was detected in regions 5 μm away from the same fAuNP on a dendrite. The amplitude and length of the Ca^2+^ transients were very similar for each of the 5 stimulation pulses (Fig. 2c–e). The results indicate that NALOS can be applied to fAuNPs, enabling the induction of local and repeated Ca^2+^ spikes (10 neurons, 6 independent cultures).

### Electrophysiological recordings during NALOS

**Figure 3:**
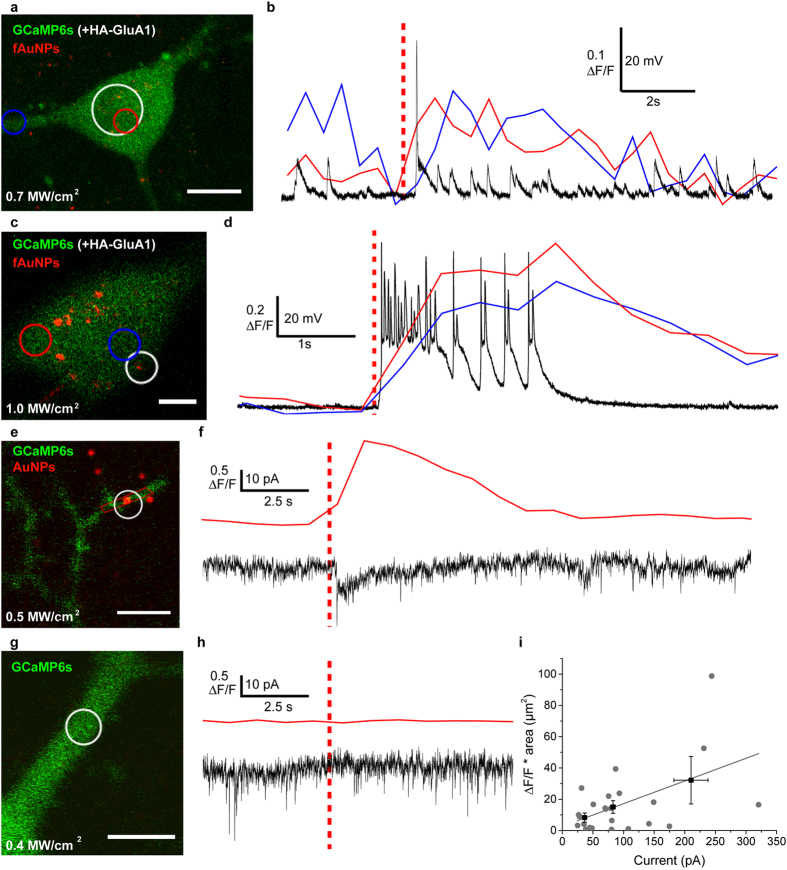
Optical and electrophysiological recording during NALOS. (**a**,**c**,**e**,**g**) Representative neurons transfected as in Fig. 2, and imaged after pre-incubation with AuNPs (**e**, or not: **g**) or fAuNPs (**a**,**c**), and simultaneously patch-clamped under whole-cell current clamp (8 neurons, 2 independent cultures) (**b**,**d**) or voltage clamp configurations (f: 24 ROIs, 11 neurons, 5 independent cultures; h: 32 ROIs, 6 neurons, 2 cultures). (**b**,**d**) Ca^2+^ signal (ΔF/F) of the blue and red regions marked in (**a**,**c**) and voltage fluctuations over the same time (black trace). At lower stimulation intensity (0.7 MW/cm^2^) (**a**,**b**), NALOS induced a single AP (distinct from spontaneous synaptic events in that neuron) while higher stimulation intensity (1.0 MW/cm^2^) induced a burst of APs (**c**,**d**). (**f**) Top trace: Ca^2+^ transient evoked by NALOS and measured in the dendritic region marked in red in (**e**); bottom trace: spontaneous miniature synaptic currents (in TTX). Note the larger current evoked by the NIR fs laser stimulus. (**h**) Top trace: in absence of AuNP, no Ca^2+^ transient (measured in the stimulation ROI [white circles in g]) was evoked by the NIR fs laser, bottom trace: no current was evoked by NALOS in absence of AuNP (in all 32 ROIs from 6 neurons tested). (**i**) Correlation between the measured Ca^2+^ and electrophysiological response. The measured ΔF/F was multiplied by the dendritic area for which a fluorescence increase above noise threshold (twice the standard deviation of the baseline) was observed. The observed Ca^2+^ response was generally higher for stronger evoked depolarization. Black squares represent the averaged (±SEM) responses for 3 grouped electrophysiological responses (25-50 pA, 50–110 pA, 110–320 pA). A linear fit of the data is shown in grey (slope 0.15 ± 0.05, Pearson’s R = 0.51) (24 ROIs, 11 neurons, 5 independent cultures). The red dashed lines shown in (**b**,**d**,**f**,**h**) indicate the time point of laser stimulation. Stimulated area is indicated with a white circle in (**a**,**c**,**e**,**g**). Scale bars (**a**) 10 μm (**c**,**e**,**g**) 5 μm. Power range for **e-i**: 0.16–0.57 MW/cm^2^.

To further characterize the mechanism of NALOS, we combined the optical stimulation and imaging with whole-cell patch clamp recordings on GCaMP6s-transfected neurons, pre-incubated for 2h with AuNPs or fAuNPs. We first tested if an AP could be observed by stimulating a sub-region of the soma. At lower intensity (0.7 MW/cm^2^), a single pulse indeed could induce an AP, which was correlated with a widespread but small Ca^2+^ transient (Fig. 3a,b). Stimulation at higher intensity (1 MW/cm^2^) induced a stronger widespread Ca^2+^ transient and was characterized by a burst of APs (Fig. 3c,d).

We next tested whether the localized Ca^2+^ response observed in dendrites with NALOS was dependent on the generation of an AP, and whether any membrane current was evoked by NALOS. AP generation was inhibited using tetrodoxin (TTX, 0.5 μM) but miniature post-synaptic currents remained. To induce a local response, we applied a laser scan of 0.4 – 0.5 MW/cm^2^ (800 nm) in a small ROI around a AuNP on a proximal dendrite (Fig. 3e–h). One scan over the ROI was sufficient to induce a membrane depolarization that was correlated to a Ca^2+^ response localized to a region of 14.5 μm^2^. Using a laser intensity in the range of 0.16–0.57 MW/cm^2^ we could correlate the observed localized Ca^2+^ response with the recorded currents (Fig. 3i) (24 local stimulations, 11 neurons, 5 independent cultures). In presence of TTX, no reversible widespread Ca^2+^ was observed and the evoked currents ranged from 25–320 pA. By contrast, the same laser stimulation on a region without AuNPs induced neither a Ca^2+^ response nor a time-correlated membrane depolarization, although spontaneous synaptic activity could be measured (Fig. 3g,h) (32 local stimulations, 6 neurons, 2 independent cultures). These results indicate that NALOS on large ROIs on the cell body can be used to drive APs. Yet, NALOS on dendritic compartment was also able to drive local and small currents capable of inducing localized Ca^2+^ responses in presence of TTX.

### NALOS to investigate localized Ca^2+^ signaling

**Figure 4:**
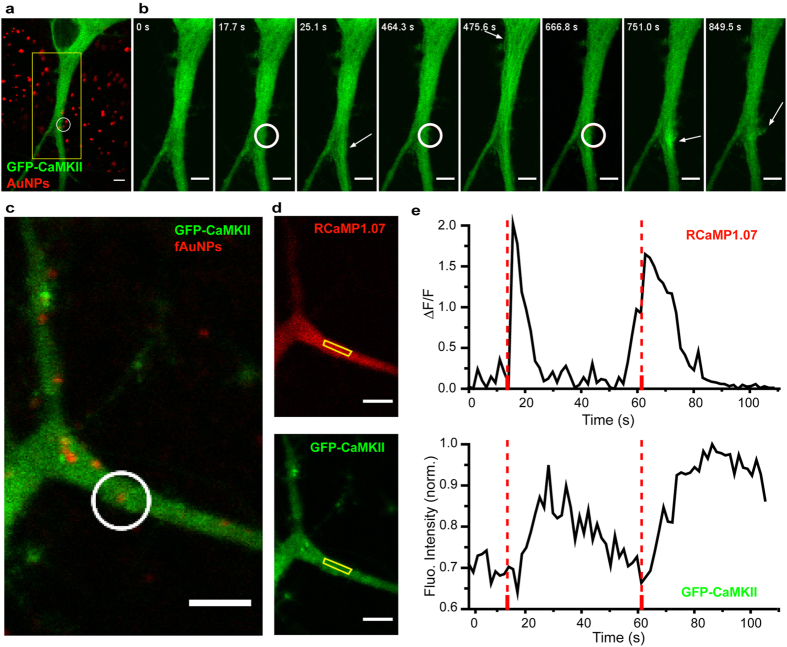
CaMKII translocation to local dendritic domains stimulated by NALOS. (**a**) Fluorescence of GFP-CaMKII (green) and reflectance of the AuNPs (red) before stimulation. (**b**) Magnification of the region marked in (**a**) for 8 different time points. The white circle shows the region of NALOS and the white arrows mark the region where GFP-CaMKII translocation was observed. Representative example of 18 neurons. (**c**,**d**) Neuron co-transfected with GFP-CaMKII and RCaMP1.07. (**c**) Fluorescence of GFP-CaMKII and reflectance of the fAuNPs before stimulation. (**d**) Fluorescence of RCaMP1.07 (top) and GFP-CaMKII (bottom) 1.5 s after the first stimulation. (**e**) Top trace: Ca^2+^ levels measured in the yellow region marked in (**d**); bottom trace: Intensity of GFP-CaMKII in the same region. Representative example of 6 neurons. Note the spontaneous Ca^2+^ transient that precedes the 2^nd^ stimulus, triggering a longer lasting Ca^2+^ event and CaMKII response. White circle in (**c**) shows the region of photo-stimulation at the time points marked with dotted lines in (**e**). Scale bars 5 μm.

The capacity to induce localized Ca^2+^ transients in somato-dendritic regions of neurons with NALOS prompted us to examine whether this approach could also serve to investigate local downstream signaling from Ca^2+^. The enzyme CaMKII is a decoder of Ca^2+^ oscillations^25^ and translocates dynamically to somato-dendritic domains exhibiting Ca^2+^ transients^26^. We performed NALOS on neurons expressing GFP-CaMKII^27^ and observed reversible and repetitive subcellular translocation of the enzyme (Fig. 4a,b). In dendrites of large caliber, it was possible to discern a microtubular pattern of translocation (n = 18) (Fig. 4b), as previously described by Lemieux *et al.*^26^. Occasionally, membrane outgrowth was observed after repeated stimulation at the region of the stimulation (Fig. 4b). We compared the translocation response of CaMKII with Ca^2+^ signals, measured with RGCaMP1.07 during NALOS. Low stimulation intensity (0.58 MW/cm^2^) in a small ROI triggered a rapid and localized Ca^2+^ transient accompanied by a slower and delayed translocation of GFP-CaMKII in the same region, while surrounding regions did not exhibit a Ca^2+^ transient, nor GFP-CaMKII translocation (n = 6) (Fig. 4c–e). Spontaneous activity combined with NALOS induced a longer lasting Ca^2+^ and CaMKII response (Fig. 4e, second stimulation). A similar sequence of events was observed during local synaptic activation of neurons^26^. Finally, NALOS with higher laser intensity (0.92 MW/cm^2^) stimulated a widespread Ca^2+^ response, which triggered a long lasting translocation of CaMKII to spines throughout the dendritic arborisation (Suppl. Fig. 4). These results indicate that NALOS using low laser intensity can be used to investigate local dendritic Ca^2+^ signaling.

## Discussion

In this study, we show that NALOS allows for localized stimulation of neuronal cells with high spatial and temporal precision. This was achieved by generating off-resonance excitation of single 100 nm AuNPs with a pulsed fs laser at 800 nm on small ROIs on cultured hippocampal neurons. In the absence of AuNPs, the fs laser did not induce any measurable effect on the cells. The size of the responding area appeared to be dependent on the created near-field enhancement and the resulting plasmon generation. With this method, we were able to monitor neuronal response after stimulation with two independent fluorescence detection channels, without observing any cross-talk between fluorescence excitation and AuNPs excitation. This would not have been possible with the use of a visible cw laser to illuminate AuNPs as reported previously^14^. Moreover, choosing NIR light instead of IR wavelength targeting water absorption^4^,^5^ allowed the easy coupling of the stimulation and fluorescence excitation light on a standard commercial confocal fluorescence microscope. The laser intensity needed for stimulation (0.16–1.02 MW/cm^2^) was lower than what would be necessary for two-photon fluorescence excitation and therefore no cross-talk between the off-resonance plasmonic excitation of the AuNPs and the activity read-out with Ca^2+^ imaging was observed. The use of NIR light for the excitation of the AuNPs is advantageous for combining the use of fluorescent proteins for monitoring molecular signaling. This provides an all-optical stimulation and molecular imaging approach at the subcellular level.

The functionalization of the AuNPs further increased the specificity of the method and its reproducibility by limiting substantially AuNP aggregation. It can also provide a means to target the AuNPs to specific cell types or even subcellular compartments that would express a specific transmembrane protein. We used an HA-tag antibody as proof of concept and to demonstrate specificity of the binding on transfected cells, but a specific antibody against an extracellular epitope of a native transmembrane protein could be used, avoiding the need for gene transfer. In such case, an organic rather than a genetically-encoded Ca^2+^ indicator could be combined.

Since the size of the stimulated region depends of the presence of AuNPs, it is not diffraction-limited; thus future work combining NALOS with laser scanning super-resolution imaging techniques could allow diffraction-unlimited optical stimulation and recording of neuronal signaling in nanodomains. Furthermore, it is foreseen that the method could be extended to produce highly localized phenomena, such as heat, pressure wave or nanocavitation depending of the irradiation parameters and AuNPs characteristics as described in Boulais *et al.*^20^.

NALOS provides a method to study Ca^2+^ signaling in any small cellular compartments, without the need for caged neurotransmitters, such as caged glutamate, which needs to be used at very high concentration (mM), leading to high costs and potential off-target effects^28^ and is limited to synaptic stimulation. The extent of the Ca^2+^ response can be tuned by the laser intensity. However, beyond the threshold intensity, the Ca^2+^ response can become widespread. The ability to locally induce Ca^2+^ transient should provide a useful approach to study Ca^2+^ signaling in various subcellular compartments of neurons or other cell types. For example, NALOS could serve to investigate the mechanisms of Ca^2+^-induced Ca^2+^ release from internal stores, Ca^2+^-dependent growth and structural remodeling, Ca^2+^-regulated local translation, Ca^2+^-dependent kinase activation, and several more. We tested the effect of inducing local Ca^2+^ transients on CaMKII signaling in neuronal dendrites, since this enzyme was shown to exhibit a dynamic translocation to dendritic domains where Ca^2+^ activity was spontaneously high^26^. Evidence suggested that this local dendritic translocation of CaMKII, mediated by a microtubular interaction, supports local synaptic plasticity^26^. The reproduction of a local dendritic translocation of CaMKII to a specifically targeted area with NALOS demonstrates the usefulness of the approach and further supports the role of CaMKII in decoding spatial and temporal patterns of Ca^2+^ signals in cells. By contrast, the production of a widespread Ca^2+^ signal with stronger local stimulation reproduced an effect observed by Rose *et al.*^29^ of widespread translocation of CaMKII to multiple spines.

Bezanilla and colleagues showed that diode laser-induced heating of a patch of cell membrane activates a measurable capacitive current^4^, a process enhanced with AuNPs, which can enable controlled membrane depolarization and generation of APs^14^. To drive repetitive APs, the authors used a high amount of AuNPs bound to the cells and applied a 1 ms on-resonance 532 nm laser stimulus over a significant portion, if not all, of the soma of dorsal root ganglion neurons. With NALOS, we also observed a small evoked current, likely also a capacitive one. We did not attempt to produce repetitive APs with 800 nm fs laser pulses, however with laser scanning over a small somatic area, it was possible to induce one or few APs, depending on the intensity. The effectiveness of the stimulation to drive APs varied with the ROI dimensions and the movement of the AuNPs inside the ROI. Our results suggest that instead of scanning over sub-cellular regions with a strongly focussed laser beam, photo-activation of the entire neuronal soma using a lower NA objective and lower intensity 800 nm light might also serve to drive APs at controlled frequency in neurons.

NALOS could serve as a complement to several light-dependent methods to control neuronal activity and cell signaling. It may be used in non-genetically altered cells, taking advantage of functionalization, or combined with genetically-encoded targets to select the cell type to be studied. The probably most useful feature is the capacity to target small subcellular domains for photo-stimulation at reasonably low light regime in NIR range. In addition to Ca^2+^ signaling, other local activity-dependent processes might also be available for investigation with this method.

## Methods

### Rat hippocampal cultures, plasmids, and transfection

The preparation of cultured dissociated hippocampal neurons was described previously^27^,^30^. Before dissection of hippocampi, neonatal rats were sacrificed by decapitation, in accordance to the procedures approved by the animal care committee of Université Laval. The dissociated cells were plated on poly-d-lysine-coated glass coverslips (12 or 18 mm) at a density of 4225 or 860 cells/mm^2^ respectively. Growth media consisted of Neurobasal and B27 (50:1), supplemented with penicillin/streptomycin (50 U/mL; 50 μg/mL) and 0.5 mM L-GlutaMAX (Invitrogen). Fetal bovine serum (2%; Hyclone) was added at time of plating. After 5 days, half of the media was changed without serum and with Ara-C (5 μM; Sigma-Aldrich) to limit proliferation of non-neuronal cells. Twice a week thereon, half of the growth medium was replaced with serum- and Ara-C–free medium. The neurons were transfected at 11–14 days *in vitro* (DIV) with Lipofectamine 2000 (Invitrogen) as described previously^27^. For Ca^2+^ imaging, plasmids encoding GCaMP6s^21^ and RCaMP1.07 (gift of J. Nakai) were transfected 1 day prior to measurements. CaMKII was tagged with a monomeric GFP as described previously^27^.

### Confocal imaging and laser-induced stimulation

NALOS and confocal imaging were performed on a confocal laser scanning microscope (LSM 510, Zeiss). Neurons were imaged in HEPES-aCSF (pH 7.4) using a perfusion system with temperature adjusted to 29–30 °C, either an open chamber (for simultaneous electrophysiological recording) or closed custom-made superfusion imaging chamber. GCaMP6s or RGCaMP1.07 were used as probes for Ca^2+^ imaging. GCaMP6s and GFP-CaMKII were excited with a continuous wave 488 nm Argon laser and detected with a 500–530 nm bandpass filter. RGCaMP1.07 was excited with a continuous wave 543 nm Helium-Neon laser and detected with a 565–615 nm bandpass filter. Off-resonance plasmonic excitation of the AuNPs was achieved with a fs pulsed Ti:Sapphire laser at 800 nm with a diffraction-limited PSF (90 MHz, 140 fs, Chameleon, Coherent). For the experiments combining NALOS and current clamp recording, a similar LSM confocal microscope was used with a 80 MHz pulsed Chameleon Ti:Sapphire laser at 800 nm. The power of the laser after the objective was tuned between 0.5 and 2 mW, which corresponds to a focal spot intensity of 0.27–1.02 MW/cm^2^ when using a 0.8 NA water immersion objective. When using 18 mm glass coverslip combined with a closed perfusion chamber, a 1.0 NA oil immersion objective was used and the laser intensity was adjusted to obtain the same focal intensity. For NALOS, a single scan over a ROI was performed with a pixel dwell time varying between 1.3 and 6.4 μs. The excitation of GCaMP6s with a laser at 488 nm had no measurable effect on the neurons with AuNPs at the imaging intensity of 0.2–0.9 kW/cm^2^.

Imaging of the AuNPs was performed in reflectance mode by taking advantage of the small transmittance of the excitation filter at 800 nm. The reflected light was detected using a long pass filter (LP 560). With this method, the position and the movement of the AuNPs could be monitored during the entire experiment. The resulting power of the 800 nm fs laser at the back aperture of the objective was less than 100 nW and therefore insufficient to generate AuNP-induced stimulation. However, when imaging RGCaMP1.07 and GFP-CaMKII, a band pass filter (BP565–615) blocking 800 nm light was used for the red detection channel and therefore the reflection of the AuNPs could not be detected during the fluorescence measurements. In this case, mapping of the AuNPs positions was performed prior to the measurement.

### Functionalization of the gold nanoparticles

Rat monoclonal anti-HA antibodies (clone 3F10, 150 kDa, Roche Diagnostics) were tethered to AuNPs using heterobifunctional PEG linkers by using the strong covalent binding between sulfur and gold^22^,^31^,^32^. The anti-HA antibodies were added to a solution of orthopyridyl disulfide-PEG-*N*-hydroxysuccinimide (OPSS-PEG-NHS, 5kDa, Nanocs) in aqueous Na_2_CO_3_ 10 mM pH 8.5 (100 μg/mL, antibodies/OPSS-PEG-NHS molar ratio: 1:1.89). The PEG-antibodies solution was mixed by vortexing and kept at 4 °C during 3h. Aliquots of the solution were kept at −20 °C until use. fAuNPs were prepared following our previously reported procedure^22^. An aqueous solution of citrate-coated AuNPs (2 mL) was treated with Na_2_CO_3_ 10 mM pH 8.5 (212 μL) and the solution of PEG-antibodies (10 μL) at 4 °C during 1h. Then, an aqueous solution of 50 μM PEG-SH (5 kDa, 247 μL, Nanocs) was added to the suspension to block the remaining free sites on the Au surface at 4 °C during 1h. The suspension was purified by centrifugation at 5000 rpm during 2 min. The supernatant was removed and replaced with Neurobasal and B27 (50:1) or phenol red-free Dulbecco’s Modified Eagle’s Medium (DMEM, Invitrogen). Aliquots of the unpurified or purified mixtures in DMEM (320 μL) were treated with water or 10% NaCl (35.5 μL) at 4 °C during 30 min and analyzed by UV-visible-NIR spectroscopy in a 96-well plate. The absorption spectra (400 to 800 nm, 2 nm step) were acquired with an Epoch microplate spectrophotometer (BioTek Instruments) controlled with the Gen5 Data Analysis software version 1.11.5. A blank spectrum (solvent without NPs) was subtracted from each sample spectrum.

### Gold nanoparticle incubation

Prior to imaging, incubation for 2h of the AuNPs onto the neurons inside the incubator allowed for sufficient sedimentation^33^, while incubation 90 min was sufficient for specific binding of the fAuNPs. For AuNPs passive sedimentation, 50 μL of the stock solution (50 μg/mL, 100 nm diameter, Nanopartz, A11-100-CIT-100, 5.71 × 10^9^ AuNPs/mL, ε = 1.1 × 10^11^ M^−1^cm^−1^, potential zeta −46 mV) was mixed with 50 μL of neurobasal cell culture medium and added dropwise to the cells. For specific binding, fAuNPs solution was centrifuged at 2000 rpm during 2 min and the fAuNPs were resuspended in neurobasal culture medium. 100 μL of this solution was then added to the cells for 1 to 2 h incubation. Longer incubation times increased non-specific binding. The AuNPs and fAuNPs solutions were kept at 4 °C and experiments with fAuNPs were performed within 2 weeks after fAuNPs synthesis.

### Electrophysiological recording

Whole-cell voltage-clamp and current-clamp recordings were obtained from visually identified pyramidal cells. For recordings, the glass pipettes of 3.5–5 MΩ were filled with a solution containing the following (in mM): 96 CsMeSO_3_, 20 CsCl, 10 diNa-phosphocreatine, 10 HEPES, 2.5 MgCl_2_, 0.6 EGTA, 4 ATP-Tris, 0.4 GTP-Tris, pH 7.28; 245 mOsm/L for voltage clamp and 111 KMeSO_3_, 10 diNa-phosphocreatine, 10 HEPES, 2.5 MgCl_2_, 2 ATP-Tris, 0.4 GTP-Tris, pH 7.25; 240 mOsm/L for current clamp. Data acquisition (filtered at 1.8–2 kHz and digitized at 10 kHz) was performed using a Multiclamp 700B amplifier and the Clampex 10.6 software (Molecular Devices). Data were analyzed using Clampfit 10.2 (Molecular Devices) and Igor Pro (WaveMetrics).

### Ca**2+** imaging analysis

Analysis of the Ca^2+^ fluctuations was done as described previously^26^ to obtain background corrected ΔF/F fluorescence traces. For the correlation with electrophysiological measurements, the measured maximal value of ΔF/F was multiplied by the dendritic area for which a fluorescence increase above noise threshold (twice the standard deviation of the baseline) was observed. The area of fluorescence increase was measured using the software Fiji^34^ and data analysis was done with the software Origin2015.

## Additional Information

**How to cite this article**: Lavoie-Cardinal, F. *et al.* Gold nanoparticle-assisted all optical localized stimulation and monitoring of Ca^2+^ signaling in neurons. *Sci. Rep.* **6**, 20619; doi: 10.1038/srep20619 (2016).

## Supplementary Material

Supplementary Information
