# Supplementary material for: Gold nanoparticle-assisted all optical localized stimulation and monitoring of Ca2+ signaling in neurons
Source: Sci Rep. 2016 Feb 9;6:20619. doi: 10.1038/srep20619 (PMC4746645; doi:10.1038/srep20619)
Supplement: Supplementary Information [file srep20619-s1.docx]

Gold nanoparticle-assisted all optical localized stimulation and monitoring of Ca^2+^ signaling in neurons

Flavie Lavoie-Cardinal^1^, Charleen Salesse^1^, Éric Bergeron^3^, Michel Meunier*^,3^, Paul De Koninck*^,1,2^

^1^ Institut Universitaire en Santé Mentale de Québec, 2601 de la Canardière, Québec, QC, G1J 2G3, Canada

^2^ Département de Biochimie, Microbiologie et Bio-informatique, Université Laval, Québec, QC, G1V 0A6, Canada

^3^ Laser Processing and Plasmonics Laboratory, Engineering Physics Department, École Polytechnique de Montréal, Montréal, QC, H3C 3A7, Canada

* co-principal investigators email: [michel.meunier@polymtl.ca](mailto:michel.meunier@polymtl.ca), [paul.dekoninck@neurosciences.ulaval.ca](mailto:paul.dekoninck@neurosciences.ulaval.ca)


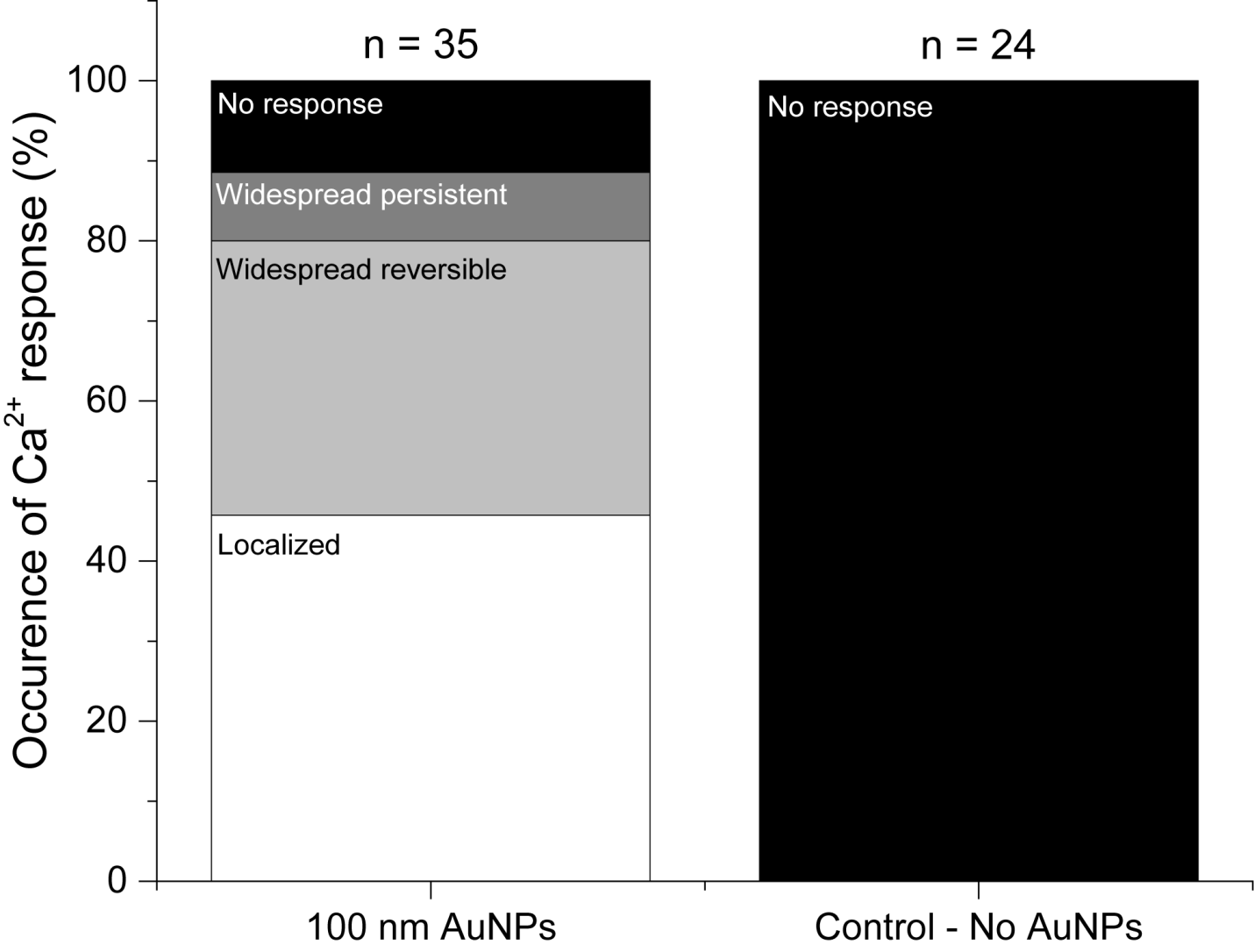


**Supplementary Figure 1: Characterization of the observed Ca^2+^ response after NALOS.**

The fluorescence increase of GCaMP6s following illumination with the 800 nm fs laser over a small region (1-6 µm^2^) was characterized in neurons incubated with 100 nm AuNPs (35 neurons, 3 independent cultures) and control neurons without AuNPs (24 neurons, 5 independent cultures). Three trials per neuron were allowed for both conditions and in case of multiple responses the first registered response was considered. When incubated with AuNPs, 80% of the neurons showed a reversible Ca^2+^ response. The control neurons without AuNPs did not show measureable GCaMP6s response for the intensity range used in NALOS. A Ca^2+^ response was considered when the ΔF/F raised to more than twice the standard deviation of the baseline trace after the fs laser illumination. The laser intensity at the focal point was in the range : 0.36-0.71 MW/cm^2^.

**Supplementary Figure 2: UV-visible-NIR spectra of bare AuNPs and fAuNPs with PEG and monoclonal anti-HA antibodies.** Samples before purification or after centrifugation and resuspension in phenol red-free DMEM were incubated for 30 min in water or 1% NaCl. Five other independent experiments gave similar results.


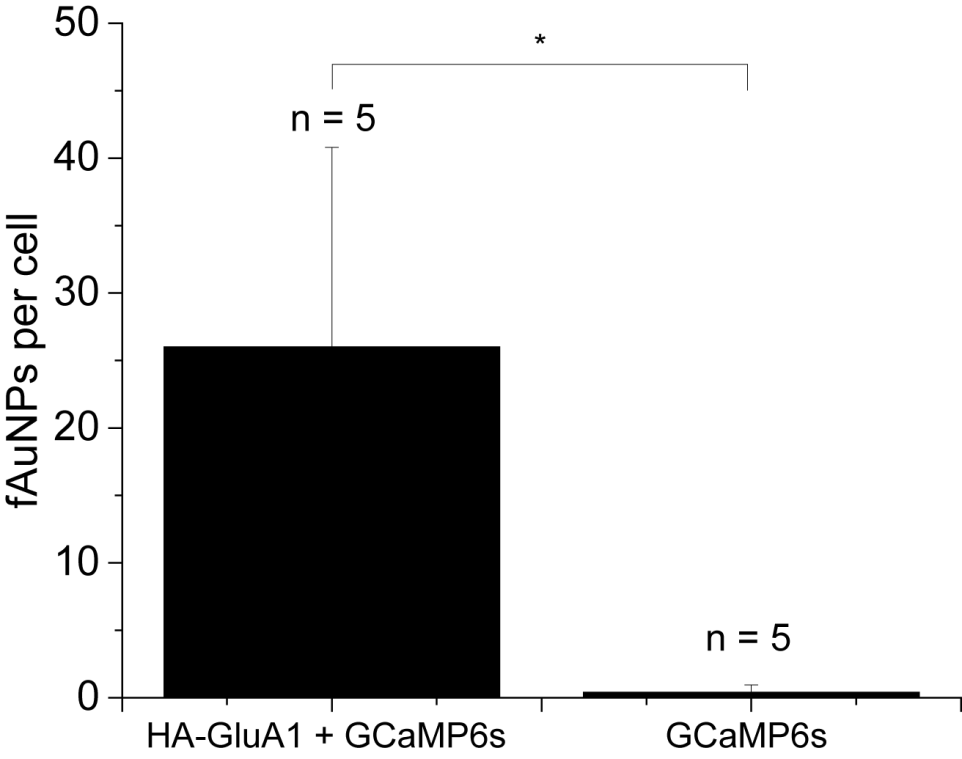


**Supplementary Figure 3: Characterization of the specificity of fAuNPs on transfected hippocampal neurons.** The number of detected fAuNPs was characterized for neurons transfected with HA-GluA1 and GCaMP6s and compared with neurons that were transfected only with GCaMP6s, lacking the HA-tag for fAuNPs binding. For the HA-positive neurons, the number of fAuNPs detected in the imaged field of view was between 13 and 49, while it was between 0 and 1 for HA-negative neurons. (n = 5 for each group). These two groups are significantly different (p < 0.05, Mann-Whitney test).


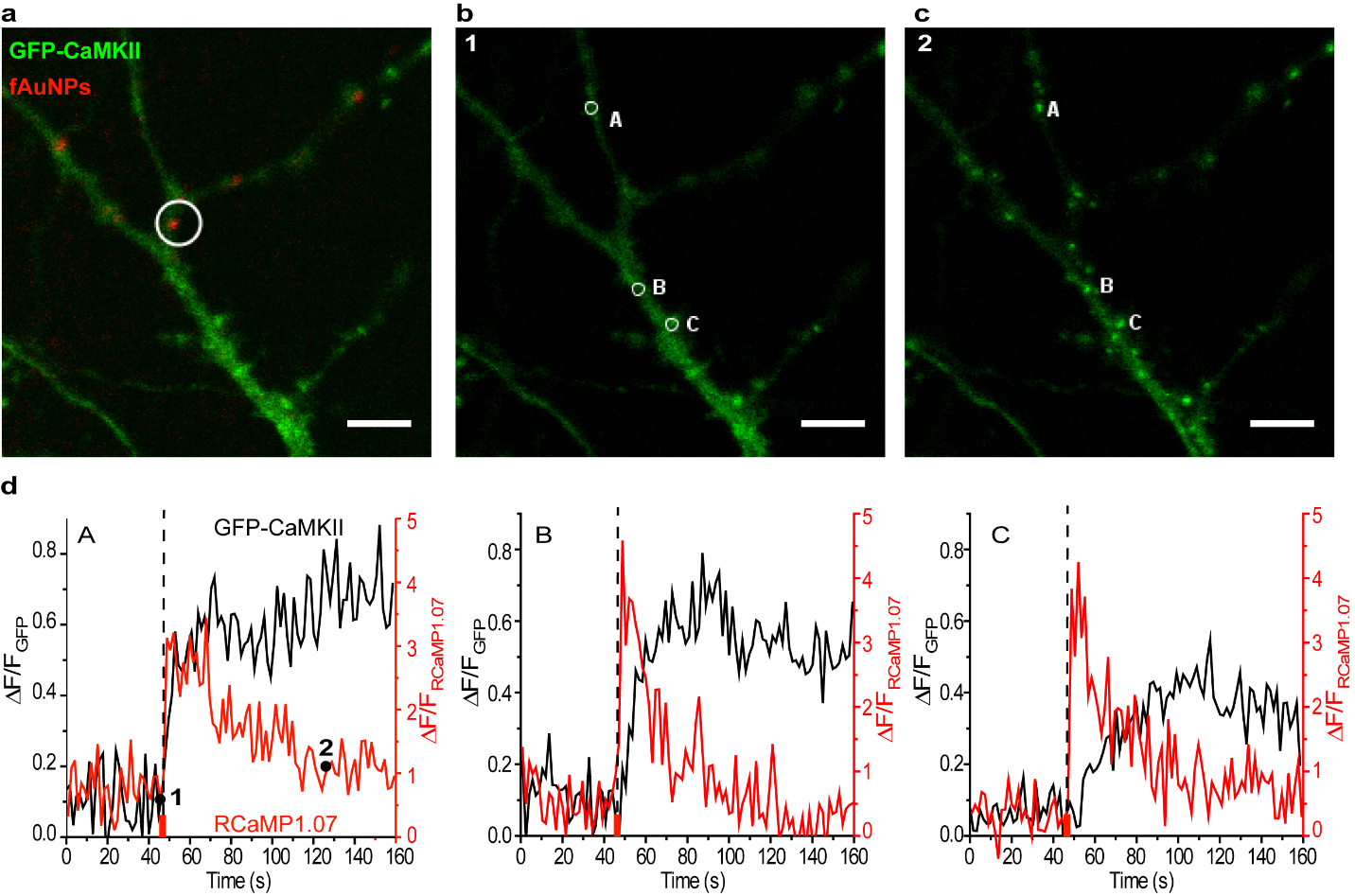


**Supplementary Figure 4: Widespread Ca^2+^ transient following high intensity stimulation in localized dendritic region leads to widespread CaMKII translocation to spines.** (a) Fluorescence of GFP-CaMKII (green) and 800 nm reflectance of AuNPs (red) before stimulation, with the high intensity (0.92 MW/cm^2^) stimulation in the white circle. (b-c) Fluorescence of GFP-CaMKII before (b) and after stimulation (c) showing persistent CaMKII translocation to the spines. (d) ∆F/F traces of the GFP-CaMKII fluorescence (black) and of the Ca^2+^ indicator RCaMP1.07 (red) of the regions marked in (b). Points 1 and 2 correspond to the time point of the images shown in (b) and (c) respectively. The stimulation time point is marked with a black dashed line. Scale bar 5 µm.

**Supplementary Table 1: Plasmon peak of bare AuNPs, PEGylated AuNPs and fAuNPs measured by UV-visible-NIR spectroscopy.** The samples before purification or after centrifugation and resuspension in phenol red-free DMEM were incubated for 30 min in water or 1% NaCl (n = 6).

|  | **Before purification** | | | **After purification** | | |
| --- | --- | --- | --- | --- | --- | --- |
|  | Bare AuNPs | PEGylated AuNPs | fAuNPs | Bare AuNPs | PEGylated AuNPs | fAuNPs |
| Water | 565.0 ± 0.6 | 566.8 ± 0.4 | 568.3 ± 0.8 | 577.2 ± 8.5 | 566.8 ± 0.4 | 568.3 ± 0.5 |
| 1% NaCl | 561.0 ± 5.1 | 567.0 ± 0.0 | 568.5 ± 0.5 | 574.5 ± 6.3 | 567.7 ± 0.8 | 569.0 ± 0.6 |
